# Natural Cocoons Enabling Flexible and Stable Fabric Lithium–Sulfur Full Batteries

**DOI:** 10.1007/s40820-021-00609-3

**Published:** 2021-03-06

**Authors:** Yanan An, Chao Luo, Dahua Yao, Shujing Wen, Peitao Zheng, Shangsen Chi, Yu Yang, Jian Chang, Yonghong Deng, Chaoyang Wang

**Affiliations:** 1 Research Institute of Materials Science, South China University of Technology, Guangzhou, 510640 China; 2 Department of Materials Science and Engineering, Guangdong Provincial Key Laboratory of Energy Materials for Electric Power, Southern University of Science and Technology, Shenzhen, 518055 China; 3 College of Materials and Energy, Guangdong Laboratory for Lingnan Modern Agriculture, South China Agricultural University, Guangzhou, 510642 China; 4 Academy for Advanced Interdisciplinary Studies, Southern University of Science and Technology, Shenzhen, 518055 China

**Keywords:** Lithium–sulfur batteries, Flexible batteries, Carbonized silk fabric, Lithium dendrite, Shuttle effect

## Abstract

**Highlights:**

- A creative cooperative strategy involving silk fibroin/sericin is proposed for stabilizing high-performance flexible Li–S full batteries with a limited Li excess of 90% by simultaneously inhibiting lithium dendrites, adsorbing liquid polysulfides, and anchoring solid lithium sulfides.
- Such fabric Li–S full batteries offer high volumetric energy density (457.2 Wh L^−1^), high-capacity retention (99.8% per cycle), and remarkable bending capability (6000 flexing cycles at a small radius of 5 mm).

**Abstract:**

Lithium–sulfur batteries are highly appealing as high-energy power systems and hold great application prospects for flexible and wearable electronics. However, the easy formation of lithium dendrites, shuttle effect of dissolved polysulfides, random deposition of insulating lithium sulfides, and poor mechanical flexibility of both electrodes seriously restrict the utilization of lithium and stabilities of lithium and sulfur for practical applications. Herein, we present a cooperative strategy employing silk fibroin/sericin to stabilize flexible lithium–sulfur full batteries by simultaneously inhibiting lithium dendrites, adsorbing liquid polysulfides, and anchoring solid lithium sulfides. Benefiting from the abundant nitrogen- and oxygen-containing functional groups, the carbonized fibroin fabric serves as a lithiophilic fabric host for stabilizing the lithium anode, while the carbonized fibroin fabric and the extracted sericin are used as sulfiphilic hosts and adhesive binders, respectively, for stabilizing the sulfur cathode. Consequently, the assembled Li–S full battery provided a high areal capacity (5.6 mAh cm^−2^), limited lithium excess (90%), a high volumetric energy density (457.2 Wh L^−1^), high-capacity retention (99.8% per cycle), and remarkable bending capability (6000 flexing cycles at a small radius of 5 mm).
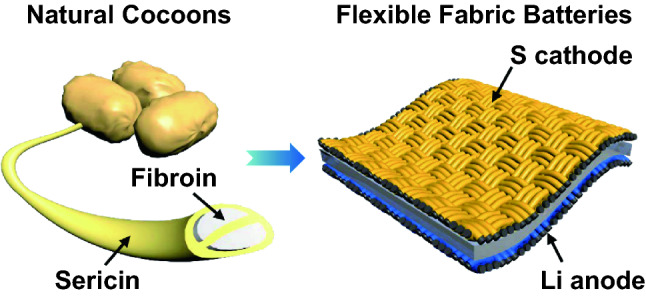

**Supplementary Information:**

The online version contains supplementary material available at 10.1007/s40820-021-00609-3.

## Introduction

Emerging flexible and wearable electronics, such as roll-up displays, bendable phones, wearable heaters, smart-watch belts, and medical patches, have gradually changed the way people live and think in recent years. Further paradigm shifts toward flexible and wearable products have imposed unprecedented demand for the seamless integration of flexible electronic devices with intrinsically flexible batteries [1–6]. Lithium-ion batteries based on intercalation chemistries dominate the current battery technology for wearable and portable electronics, but have reached the limit of their theoretical energy density. Lithium–sulfur (Li–S) batteries are considered promising alternatives for traditional Li-ion batteries because of the low mass densities (Li: 0.534 g cm^−3^; S: 2.07 g cm^−3^) and high theoretical capacities (Li: 3,860 mAh g^−1^; S: 1675 mAh g^−1^) of their components, and their overall high energy density (2,600 Wh kg^−1^) [7–12]. Despite these remarkable advantages, most of the currently reported Li–S batteries still require the use of heavy Li-foil anodes (~ 50 mAh cm^−2^) and face several hazards as follows: 1) low Coulombic efficiency (CE) both in the Li anode and S cathode due to the easy formation of Li dendrites, shuttle effect of dissolved lithium polysulfides (Li_2_S_*x*_, 3 ≤ *x* ≤ 8), and random deposition of insulating Li_2_S; 2) large volume expansion of the Li anode (infinite) and S cathode (~ 80%) during the cycling process; and 3) poor mechanical flexibility of the electrodes during the flexing process [8, 13]. Therefore, it is highly desirable to realize high-energy–density, flexible, and stable Li–S full batteries with limited Li excess by rationally designing both the Li and S electrodes [1, 14].

To overcome the above-mentioned challenges, many strategies have been proposed for stabilizing the Li anode and S cathode with high CEs, including the modification of the solid electrolyte interface (SEI) [15–18], the introduction of solid-state electrolytes [19, 20], and the development of three-dimensional (3D)-structured hosts [1, 21, 22]. In contrast, 3D-structured hosts with unique surface chemistry and interconnecting structures are more effective for stabilizing Li and S electrodes by mitigating their volume expansion and regulating the uniform distribution of Li ions. In particular, 3D carbon hosts (such as carbon fabrics, graphene, and CNT-derived papers) have been widely developed for constructing Li anodes and S cathodes due to their easy accessibility, superior thermal stability, and excellent flexibility [14, 23]. However, these non-polar carbon hosts have poor affinity for polar Li metal and dissolved polysulfides [24–27], resulting in loss of both electrode materials and rapid capacity fading during cycling processes. To enable the uniform deposition of Li metal and strong anchoring of polysulfides and Li_2_S, heteroatom doping (e.g., N, P, O, S, and B) [24, 28, 29], introduction of metal compounds (e.g., MnO_2_, MoS_2_, and VN) [30–33], and metal (e.g., Co, Ni, and Sn) [34–36] modifications have been widely applied to 3D carbon hosts to regulate their interfacial polarity. The interfacial doping of heteroatoms, especially dual-element doping, is simple and effective for stabilizing both electrodes during long-term cycling because of the strong and reversible interactions of the heteroatoms with metallic Li and Li_2_S_*n*_. In previous simulation studies, N/O dual-doped carbon hosts were predicted to be the most effective for the uniform deposition of Li metal and strong anchoring of polysulfides and Li_2_S [37, 38]. Therefore, rationally designed N/O dual-doped carbon hosts are promising candidates for the successful fabrication of flexible Li–S full batteries.

Apart from the cycling stabilities of the Li anode and S cathode, both electrodes must be able to withstand large mechanical strain to realize flexible Li–S batteries. It is recognized that the strain (*ɛ*) applied to the cell components of a matched flexible battery for industrial flexible electronics devices is typically larger than 5% [20, 39]. Commercial carbon fabric with larger mechanical strain resistance (> 10%) and a controllable structure compared to available carbon (CNT and graphene) papers has attracted extensive research interest for its potential use in flexible Li–S batteries. For example, the first flexible Li–S full battery devices with limited Li excess have recently been fabricated by using metal-coated carbon fabrics as current collectors [1]. Such fabric-type Li–S full batteries exhibit excellent cycling and mechanical stabilities, but still suffer from limited cell energy density owing to the large areal mass and low surface area of the used carbon fabrics [1, 39]. Since natural silk cocoons are composed of fibroin and sericin, carbonized fibroin fabric has been widely investigated for its application in bendable and stretchable electronic devices due to its adjustable areal mass, adequate mechanical strain, and high electrical conductivity [40, 41]. However, to the best of our knowledge, carbonized fibroin fabric or extracted sericin protein (SP) have not been explored for the successful fabrication of flexible Li–S batteries.

In this paper, a cooperative strategy employing silk fibroin/sericin is proposed for stabilizing flexible Li–S full batteries with a limited Li excess of 90%, exceptional mechanical flexibility, high volumetric energy density, and excellent cycling stability. The uniform deposition of Li metal and Li_2_S by loading Li or S onto ultrathin and soft N/O-codoped carbonized fabric (NOCF) was crucial in developing these batteries. In particular, silk-fibroin-derived NOCF is used as a lithiophilic host for the Li anode, while NOCF and SP are used as sulfiphilic hosts and adhesive binders, respectively, for the S cathode. The unique fabric structure of NOCF simultaneously provides mechanical flexibility and reduces the local current density of the electrodes. Importantly, NOCF could also stabilize both electrodes to reach remarkable CEs. On the anode side, NOCF could ensure uniform deposition of Li nanoparticles instead of dendrites and lead to a high average CE > 99.4% over 300 charge/discharge cycles. On the cathode side, NOCF could strongly adsorb dissolved polysulfides and promote the uniform anchoring of solid Li_2_S, resulting in an excellent capacity retention of > 99.9% per cycle for 200 cycles. Finally, mechanically robust Li–S full cells were successfully obtained with a high volumetric energy density (457.2 Wh L^−1^), high areal capacity (5.6 mAh cm^−2^), and excellent cycling stability (capacity retention per cycle: > 99.8%). The Li–S full batteries could also maintain stable charge/discharge characteristics over 150 cycles during dynamic flexing processes and power the displays of large light-emitting diode (LED) screens for tens of minutes, even when repeatedly bent over 6,000 flexing cycles at a small radii of curvature (5.0 mm).

## Experimental Section

### Preparation of Fabric Electrodes

#### Preparation of N/O-Codoped Carbonized Silk Fabric

The soft NOCF current collectors were prepared by direct carbonization of commercial silk fabric in an inert atmosphere of Ar gas by a gradient heating method. First, commercial silk fabric was rinsed with deionized water and ethanol several times under ultrasonic conditions and dried at 60 °C for 5 h. Second, the dried silk fabric was placed in a tube furnace and carbonized in a high-purity argon atmosphere (purity: 99.999%; gas flow: 120 sccm) with the following carbonization procedure: (1) heat from 25 to 150 °C at a rate of 5 °C min^−1^ and maintain the temperature at 150 °C for 0.5 h; (2) heat to 350 °C at a rate of 3 °C min^−1^ and maintain the temperature at 350 °C for 2 h; (3) heat to 1,000 °C at a rate of 2 °C min^−1^ and maintain the temperature at 1,000 °C for 1.5 h; and (4) naturally cool the system to room temperature. Finally, the achieved carbonized silk fabric was rinsed with deionized water and ethanol several times and dried at 60 °C for 12 h. In addition, heating at various carbonization temperatures was also performed to reveal the impact of temperature on the electrical conductivity and elastic strain of the carbonized silk fabrics.

#### Preparation of Flexible Fabric Lithium Anode

The NOCF was cut to a certain size and shape and served as the working electrode in the sandwiched cell, while polypropylene fabric was used as the separator and lithium foil served as the counter/reference electrode. The prepared electrolyte was 1 M lithium bis(trifluoromethanesulfonyl)imide (LiTFSI) in a mixture of 1,3-dioxolane (DOL) and 1,2-dimethoxyethane (DME) (1:1, v/v) with 2 wt% of a LiNO_3_ additive. The cell of NOCF versus Li foil was first cycled at 0–2.0 V at 1.0 mA cm^−2^ for five cycles and then discharged to form the Li/NOCF anode until the required Li metal was deposited. The Li/CF and Li/Cu reference anodes were prepared using the same procedure.

#### Preparation of Sericin Protein (SP) Binder

A neutral hydrothermal strategy was adopted for extracting the high-molecular-weight SP from natural cocoons by relieving sericin hydrolysis. Natural silkworm cocoons were first cut into small pieces, rinsed with deionized water and ethanol three times, and then dried at 60 °C. Second, the cocoons soaked in deionized water (1:100, w/v) were boiled for 2 h under normal pressure. Third, the formed dispersion was filtered to obtain the SP solution after the removal of the residual silk fibroin. Finally, the SP solution was dialyzed with a dialysis bag (molecular weight cut-off = 10,000) and freeze-dried for 24 h to obtain the SP powder.

#### Preparation of Flexible Fabric Sulfur Cathode

The sulfur/carbon composite was prepared according to the traditional melt-diffusion strategy [1]. Commercial sulfur powder and Ketjen black (ECP-600JD) nanoparticles were ground together in an optimal weight ratio of 3:1. This sulfur hybrid was then heated to 155 °C in a 100-mL Teflon-lined stainless-steel autoclave and kept for 16 h. Then, the sulfur hybrid and SP binder, in a weight ratio of 9:1, were dissolved in a certain amount of deionized water and ball-milled for 30 min to produce a homogeneous sulfur-containing slurry. Finally, the sulfur/carbon/SP slurry was uniformly doctor-bladed onto NOCF and dried at 60 °C in a vacuum oven for 12 h to obtain the desired fabric sulfur cathode.

### Morphology and Structure Characterization

The surface morphology of the samples was examined by field-emission scanning electron microscopy (FESEM, Tescan MIRA3, Czech Republic). The microstructures of the samples were investigated by high-resolution transmission electron microscopy (HRTEM, Oxford INCA 200, Oxford Instruments, UK). The carbonized silk fabric sample was ball-milled for 30 min, sonicated in ethanol for 5 min, and the suspension was then dropped in a 200-mesh Cu grid. The carbonization degree of the samples was determined using a laser microscopic Raman system (RENISHAW PLC, INVIA) with an excitation energy of 2.41 eV (514 nm). The crystallographic structure of the sample was analyzed using an X-ray diffractometer (XRD, X pert pro M) with Cu Kα radiation (*λ* = 0.15406 nm). The stress–strain curve of the samples was obtained using a tensile tester (Instron 3342). The chemical structure of the samples was analyzed by Fourier-transform infrared (FTIR, Vector 33-MIR) spectroscopy and X-ray photoelectron spectroscopy (XPS, PHI 5000 Versaprobe III) with monochromatic Al Kα radiation. N_2_ adsorption/desorption analyses were conducted using a surface area analyzer (BET, TriStar II 3020 Version 3.02). The molecular weight of the sample was analyzed by gel permeation chromatography (GPC, Cirrus GPC Version 3.4.1).

### Testing for Polysulfide Adsorption and Lithium–Sulfide Nucleation

The Li_2_S_8_ catholyte (1 mM) was obtained by placing a mixture of sulfur and Li_2_S with a molar ratio of 7:1 into a DOL/DME solution (1:1, v/v) and violently stirring under an argon atmosphere at 60 °C for 24 h. Then, 20 mg of the host material of the sulfur cathode was added to the diluted Li_2_S_8_ catholyte to test the adsorption of polysulfides. To check the kinetics of Li_2_S nucleation and deposition, the host material of the sulfur cathode was paired with Li foil using a Li_2_S_8_/tetraglyme catholyte. Notably, the Li_2_S_8_/tetraglyme catholyte consists of 0.3 M Li_2_S_8_ and 1 M LiTFSI in tetraglyme. As such, the behavior of Li_2_S_8_ nucleation and deposition onto various host materials was monitored by galvanostatic discharge at 2.05 V and 0.1 mA cm^−2^ and then potentiostatic discharge at 2.04 V until the discharge current was reduced to 0.01 mA cm^−2^.

### Assembling of Fabric Lithium–Sulfur Full Batteries and Electrochemical Measurements

The fabric Li–S full cells encapsulated commercial button coin, and soft Al-plastic film was assembled in an argon-filled glove box, with the SP/S/NOCF composite as the cathode, a microporous polypropylene fabric as the separator, and a Li/NOCF composite as the anode. The electrolyte of 1 M LiTFSI in DOL/DME with 2 wt% LiNO_3_ was appropriately added according to the practical ratio (10 μL mg^−1^) of electrolyte volume to sulfur weight.

Stainless-steel coin cells and soft-packaged cells were assembled in an Ar-filled glovebox with oxygen and moisture contents < 1 ppm. In the cathodic and anodic half-cells, the electrochemical performances of the SP/S/NOCF and Li/NOCF composites were individually evaluated by galvanostatic cycling of 2025-type coin cells with the same amount of electrolyte. Galvanostatic cycling of the electrodes was conducted using a Neware battery testing system (CT2001A). Cyclic voltammetry measurements were performed on a Solarton analytical electrochemical workstation (model 1470E, England) with a voltage test range 1.7–2.8 V and a scanning rate of 0.05 mV s^−1^.

## Results and Discussion

### Design and Fabrication of Fabric Lithium–Sulfur Full Batteries

**Fig. 1:**
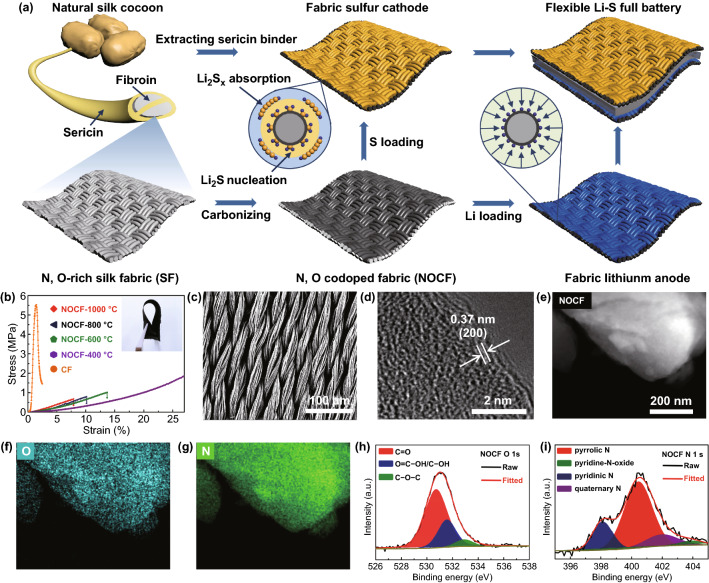
**a** Illustration of the manufacturing process and design principle of flexible Li–S full batteries. The N/O-codoped carbonized fabric (NOCF) with high flexibility, high conductivity, and abundant N/O-codoped sites is synthesized by the gradient carbonization of natural silk fabric under an inert atmosphere. Subsequent to the thermal carbonization of silk fibroin, a certain amount of Li metal was electroplated onto NOCF to yield the Li/NOCF anode and a slurry mixture containing sericin protein (SP) binder, Ketjen black, and S was coated onto NOCF to yield the SP/S/NOCF cathode. **b** Tensile stress–strain curves of CF and NOCFs at various annealing temperatures (inset shows the optical image of optimal NOCF at 1000 ℃). **c** Scanning electron microscopy (SEM) image of the optimal NOCF. **d** High-resolution transmission electron microscopy (TEM) image of the optimal NOCF. **e–g** Low-resolution TEM image (**e**) and energy-dispersive X-ray spectroscopy (EDS) elemental mapping (**f**, **g**) of the optimal NOCF. **h**, **i** High-resolution X-ray photoelectron spectroscopy (XPS) spectra of O 1*s* and N 1*s* of the optimal NOCF

Introducing a fabric-based matrix as soft current collectors has been demonstrated as an effective strategy for designing highly flexible batteries [14, 20]. To construct fabric Li–S full batteries, a soft and conductive NOCF current collector was first synthesized by the gradient carbonization of natural silk fibroin fabric in an inert atmosphere of Ar gas (Fig. 1a) [40, 42, 43]. The annealing conditions were also explored to reveal the impact of temperature on the electrical conductivity and mechanical strain of NOCF (Figs. 1b and S1). The electrical conductivity of NOCF increased with increasing annealing temperature (Table S1) due to the high degree of graphitization. Contrastingly, the elastic strain of NOCF decreased with increasing annealing temperature, but all of them exhibited much larger strain than that (1.5%) of commercial carbon felt (CF). Among the annealing temperatures tested, the NOCF exhibited the highest electrical conductivity and largest elastic strain (7.5%) at 1000 °C, which are preferred for fast electron transport and high degrees of mechanical bending (inset in Fig. 1b). In addition, the achieved NOCF has a smaller areal mass (3.0–6.0 mg cm^−2^) and thickness (100–200 μm) than those of commercial CF (13.6 mg cm^−2^, 300 μm). The obtained nitrogen adsorption and desorption isotherms of NOCF indicate that it has a specific number of micropores and macropores, along with a larger specific surface area (20.8 m^2^ g^−1^) than those of CF (16.3 m^2^ g^−1^) and graphite sheets (GSs, 11.7 m^2^ g^−1^) (Fig. S2). Due to its large specific surface area and abundant nitrogen and oxygen atoms, NOCF exhibited a much higher adsorption capability for soluble polysulfides than those provided by 3D CF and 2D lamellar GS. SEM characterization revealed the well-preserved spring structure of the twisted fibers, indicating the origin of the excellent bending capability of the Li–S fabric full batteries (Fig. 1c).

The chemical structure and composition of the NOCF are revealed by HRTEM and XPS. The TEM characterization confirms a distorted lattice fringe with an interlayer spacing of 0.37 nm, which corresponds to the interlayer spacing of the (200) plane of hexagonal graphite (Fig. 1d). The low-resolution TEM image (Fig. 1e) and corresponding energy-dispersive X-ray spectroscopy (EDS) mapping (Fig. 1f, g) demonstrate that N and O are evenly distributed at the fiber surface of NOCF. Compared to the interlayer spacing (0.33 nm) of graphite, the slightly expanded spacing of NOCF is related to the doping of N and O heteroatoms, which has been confirmed by high-resolution XPS, as shown in Fig. 1h and i. The XPS survey spectrum of NOCF confirms that the atomic contents of C, N, and O are 88.7 at%, 3.4 at%, and 7.9 at%, respectively (Fig. S3). The C 1*s* and O 1*s* spectra demonstrate the presence of C–N (285.7 eV), C–O (288.0 eV), and C=O (530.7 eV) chemical bonds (Figs. 1h and S4). In addition, the fitted N 1*s* spectrum further demonstrates four peaks of N, corresponding to pyridinic N (398.4 eV), pyrrolic N (400.3 eV), quaternary N (402.1 eV), and the N of pyridine-*N*-oxide (403.6 eV), respectively. As a result, natural silk-derived NOCF, with the advantages of high electrical conductivity, large elastic strain, and abundant N/O dual-doping sites, is a promising material for fabricating flexible Li and S electrodes.

Subsequent to the thermal carbonization of the silk fibroin, a certain amount of Li metal was electrochemically plated on NOCF to yield the Li/NOCF anode. On the other hand, a slurry mixture containing SP binder, Ketjen black, and S was coated on NOCF to yield the SP/S/NOCF cathode (Fig. 1a). Finally, the two fabric electrodes, together with a membrane separator (Celgard 2500) and ether-based electrolyte, were assembled into soft full cells and sealed with aluminum plastic film.

### Plating/Stripping Behavior and Coulombic Efficiency of the Fabric Lithium Anode

**Fig. 2:**
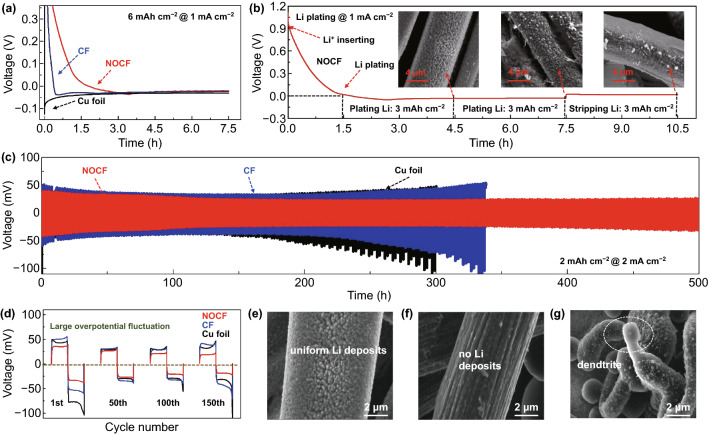
**a** First discharge voltage profiles for Li nucleation onto various hosts of NOCF, CF, and Cu foil at 1.0 mA cm^−2^. **b** Galvanostatic charge/discharge profile of NOCF (vs. Li metal) at 1.0 mA cm^−2^, and corresponding SEM images after electroplating 3.0 mAh cm^−2^ and 6.0 mAh cm^−2^ of Li and then subsequently stripping 3.0 mAh cm^−2^ of Li. **c** Galvanostatic plating/stripping profiles of symmetric cells with various Li electrodes (Li/NOCF, Li/CF and Li/Cu foil) at 1.0 mA cm^−2^. **d** Galvanostatic plating/stripping profile of various Li anodes at the 1st, 50th, 100th, and 150th cycle. **e**–**g** SEM images of Li/NOCF, Li/CF, and Li/Cu foil after plating 3.0 mAh cm^−2^ of Li metal at 1.0 mA cm^−2^

NOCF possesses an excellent capability for stabilizing Li metal during the plating/stripping process. As a proof-of-concept, we first estimated the energy barrier of Li nucleation onto NOCF by analyzing the nucleation overpotential during the first electroplating process, in which the Li nucleation overpotential is defined as the difference between the sharp tip voltage and the later, stable mass-transfer overpotential. When Li is plated onto bare Cu foil, a large nucleation overpotential of 93.7 mV is observed at a practical current density of 1.0 mA cm^−2^, which indicates an unfavorable interaction between the Cu foil and Li metal (Fig. 2a). In contrast, a much smaller nucleation overpotential of 23.2 mV is revealed for the 3D structured host of CF. Unexpectedly, the nucleation overpotential for NOCF was only 13.2 mV, which can be ascribed to two facts: (1) the large surface area of the NOCF structure facilitates the reduction in local current densities [1, 4] and (2) abundant doped sites of N/O at the fiber surface of NOCF benefit the lithiophilic nucleation of Li metal [34, 38]. Electroplating on NOCF proceeds through a two-step process: initial Li^+^ intercalation into NOCF at 0.9 V (vs. Li/Li^+^) and subsequent Li-metal deposition at the fiber surface of NOCF at nearly 0 V. It can be observed that NOCF has a lithium insertion capacity of ~ 1.5 mAh cm^−2^ at 1.0 mA cm^−2^. Therefore, in the subsequent lithium-metal deposition experiments and half-cell Coulombic efficiency tests, an additional 1.5 mAh cm^−2^ of lithium will be deposited on NOCF to compensate for the lithium inserted in NOCF, making the amount of lithium deposited on the surface of NOCF consistent with that of the control samples (CF and Cu). When the plating capacity of Li metal reaches 3.0 mAh cm^−2^, uniform Li-metal nanoparticles are clearly observed at the surface of each fiber (Figs. 2b and S5). At a plating capacity of 6.0 mAh cm^−2^, the initial small nanoparticles of Li metal became much larger and formed a thick coating layer at the fiber surface. After stripping 3.0 mAh cm^−2^ of Li metal, large particles of Li metal were restored to their original state, indicating the high reversibility of Li metal during the plating/stripping process.

Apart from the small nucleation overpotential during the first electroplating process, a low mass-transfer overpotential of Li metal is required for high CEs and long cycling stability. Here, coin cells made of one pair of Li/NOCF anodes (electrode capacity: 9.0 mAh cm^−2^) were stripped and plated in a partial capacity of 1.0 and 2.0 mAh cm^−2^ (Figs. 2c, d, and S6). At a practical current density of 1.0 mA cm^−2^, the overpotential of the Li/NOCF symmetric cell started at a very low value of ~ 20 mV and continued to decrease until reaching ~ 15 mV at the 10^th^ cycle and remained constant until reaching approximately 600 cycles (Fig. S6). When a plating capacity of 2.0 mAh cm^−2^ was applied, the Li/NOCF symmetrical cell could still maintain stable voltage profiles with a small overpotential of 25 mV for > 250 cycles at a high current density of 2.0 mA cm^−2^. It was observed that the abundant lithiophilic N/O sites of NOCF can confine the deposits of metallic Li at the fiber surface and allow the continuous formation of Li nanoparticles instead of dendrites (Figs. 2e, S7, and S8). In comparison, when one pair of anodes comprising Li on Cu foil (Li/Cu) or Li on CF (Li/CF) are assembled into symmetric coin cells, obvious dendrite formation is observed (Fig. 2f, g). In comparison, no deposits of Li metal are observed at the fiber surface of CF due to the poor affinity of Li metal to bare CF. The random deposits of Li metal observed for Li/CF led to a rapid overpotential increase and Li loss [18, 36]. For the Li/Cu foil, the uncontrolled deposits of Li dendrites induced a violent fluctuation of overpotential at the 100th cycle and a sudden jump at the 150th cycle.

**Fig. 3:**
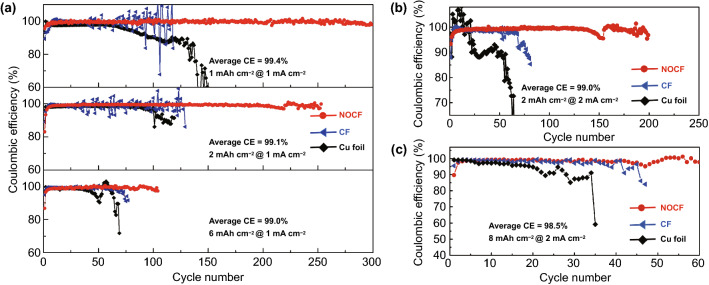
**a** Cycling stability of various Li anodes (Li/NOCF, Li/CF and Li/Cu foil) with various areal capacities of 1.0 (top), 2.0 (middle), and 6.0 (bottom) mAh cm^−2^ at 1.0 mA cm^−2^. **b**, **c** Cycling stability of various Li anodes (Li/NOCF, Li/CF and Li/Cu foil) with high areal capacities of 2.0 (**b**) and 8.0 (**c**) mAh cm^−2^ at a high current density of 2.0 mA cm^−2^

A high CE can be expected for the Li/NOCF anode due to the high stability of Li within the structured host of NOCF and the low operation overpotential discussed above. The CE of Li-metal anodes mainly depends on the stability of their SEI layer and the interfacial side reactions between the Li metal and current collectors [1]. As such, we recorded the CEs of various Li anodes, including Li/NOCF, Li/CF, and Li/Cu, against Li foil during the full plating/stripping process. Various areal capacities of Li (including 1.0, 2.0, and 6.0 mAh cm^−2^) were first fully plated and stripped at a current density of 1.0 mA cm^−2^ (Fig. 3a). With a cycling capacity of 1.0 mAh cm^−2^, a high initial CE of 95.5% was obtained in the first cycle, and an average CE of 99.4% was maintained for nearly 300 cycles. For Li/Cu, the CE rapidly dropped after only 70 cycles, resulting from dendrite formation and SEI damage. Li/CF exhibited a higher average CE than Li/Cu, indicating that a 3D fabric structure with a large surface area can significantly reduce the local current density and stabilize the SEI layer [4]. Unfortunately, Li/CF suffers from a cycling fluctuation after the 90th cycle due to the formation of Li dendrites. Impressively, even upon further increasing the cycling capacity to 2.0 or 6.0 mAh cm^−2^, the substrate of NOCF maintained a high average CE of 99.1% for 250 cycles or 99.0% for 100 cycles. With an increase in the current density to 2.0 mA cm^−2^, the NOCF also exhibits high CEs of 99.0% with a cycling capacity of 2 mAh cm^−2^ and 98.5% with a cycling capacity of 8.0 mAh cm^−2^ (Fig. 3b, c). As a result, the NOCF, with the advantages of a large-surface-area fabric structure, abundant lithiophilic N/O sites, and negligible interfacial side reactions exhibits a high cycling capacity of 8.0 mAh cm^−2^, a large current density of 2.0 mA cm^−2^, and long-term cycling stability.

### Structural and Cycling Stability of Fabric Sulfur Cathode

**Fig. 4:**
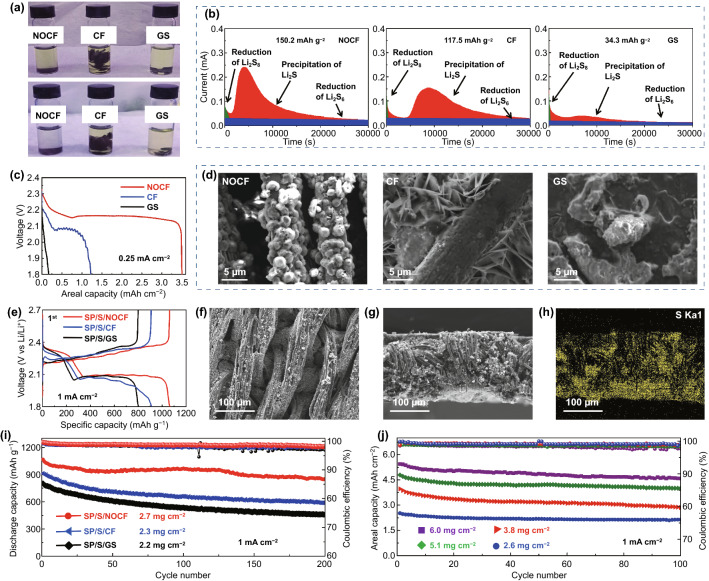
**a** Photographs showing the starting (top photo) and ending (bottom photo) states of Li_2_S_8_ adsorption onto NOCF, CF, and graphite sheets (GS). **b** Potentiostatic discharge profiles of cells with NOCF, CF, and GS. **c** Galvanostatic discharge curves of NOCF, CF, and GS (vs. Li foil) with a certain amount of Li_2_S_8_ catholyte at 0.25 mA cm^−2^. **d** Corresponding SEM images of Li_2_S deposition at the surface of various substrates, including NOCF, CF, and GS. **e** The first charge/discharge cycle of various sulfur cathodes (SP/S/NOCF, SP/S/CF, and SP/S/GS) at 1.0 mA cm^−2^. **f–h** Top-view and cross-section SEM images of SP/S/NOCF and the corresponding sulfur mapping. **i** Cycling performance of SP/S/NOCF, SP/S/CF, and SP/S/GS cathodes at 1 mA cm^−2^. **j** Cycling performance of SP/S/NOCF cathodes with various sulfur loadings

The excellent cycling stability of sulfur cathodes can be improved by the strong adsorption of dissolved polysulfides and the uniform anchoring of solid Li_2_S [24–26]. To explore the adsorption capability of NOCF for liquid polysulfides, the same amounts of various substrates (NOCF, CF, and GS) were added to the yellow solution of dissolved Li_2_S_8_ in DOL/DME for checking the chemical adsorption capability (Fig. 4a). Upon comparing the adsorption rates and amounts of Li_2_S_8_ for NOCF, CF, and GS, it was noted that NOCF adsorbed the largest amount at the fastest rate, demonstrating its effective sulfiphilic interface. Apart from the adsorption of polysulfides, the kinetics of Li_2_S nucleation and deposition were also investigated by potentiostatically discharging the Li_2_S_8_ catholyte on various host materials, including NOCF, CF, and GS (Fig. 4b). The testing cell was assembled using the host material as the working electrode and Li foil as the counter electrode. Then, the testing cell was galvanostatically discharged to 2.05 V and then potentiostatically discharged at 2.04 V. As such, NOCF exhibited a much larger peak of discharging current and a much higher capacity (150.2 mAh g^−1^) of Li_2_S deposition than those of CF (117.5 mAh g^−1^) and GS (34.3 mAh g^−1^). In addition, the kinetics and morphology of Li_2_S deposition were examined by monitoring the initial galvanostatic discharge curves (Fig. 4c) of Li_2_S_8_ with a small current density of 0.25 mA cm^−2^ and collecting the corresponding SEM images (Fig. 4d). The cell of NOCF versus Li foil exhibited a much higher capacity than the CF- and GS-containing cells, indicating that abundantly doping the fabric structure with N and O atoms offers more nucleation sites and a stronger ability for anchoring Li_2_S deposits. As shown in Figs. 4d and S9, large amounts of uniform Li_2_S nanoparticles were observed at each fiber surface of NOCF after discharging Li_2_S_8_, confirming the strong affinity of NOCF to Li_2_S. In contrast, the CF with a large-surface-area fabric structure exhibited a non-uniform deposition of Li_2_S nanosheets due to its low local current density (Figs. 4d and S9). For the planar substrate of GS, sparse and discrete deposits of Li_2_S were obtained due to the poor affinity of GS to Li_2_S (Figs. 4d and S9). As a result, the 3D fabric structure and abundant doping sites of N/O in NOCF promote the rapid kinetics of Li_2_S nucleation and deposition.

In addition to the adsorption of polysulfides and strong anchoring of insulating Li_2_S, the structural integrity of the sulfur cathode is important for long-term cycling stability [37]. Here, a sulfiphilic SP binder with abundant N/O functional groups was prepared by boiling natural silk cocoons [44, 45] and was used for inserting S and Ketjen black into NOCF. As revealed by FTIR, the SP binder with a large amount of polar N/O-containing functional groups facilitates the adsorption of polysulfides and maintains the structural stability of the sulfur cathode via hydrogen-bonding interactions (Figs. S10-S12). In addition, the adhesive strength of the sulfur cathode on the carbon-coated Al foil was also revealed by detaching the adhesive tape from the top surface of the sulfur cathode and subjecting it to nanoindentation characterization. In addition, the SP binder could provide the sulfur cathode with a much stronger binding capability and a higher Young's modulus than those of the commonly used PVDF binder (Figs. S13 and S14). Furthermore, compared to those of cathodes with the PVDF binder, the sulfur cathode with the SP binder shows much higher specific capacities and stability at a high discharging rate of 1 C (Fig. S15). As a result, the SP binder with abundant sulfiphilic sites and strong binding capability is an ideal choice for realizing stable sulfur cathodes.

To enable a flexible sulfur cathode with high capacities, a mixture slurry of SP binder, S powder, and Ketjen black additive was uniformly inserted into the fabric structure of NOCF. With the dual-polarity design of the SP binder and NOCF collector, the achieved fabric sulfur cathode, SP/S/NOCF, exhibited a satisfactory initial CE of > 99.7% after the activation process, which is much higher than those of SP/S/CF and SP/S/GS cathodes. The SP/S/NOCF cathode also shows a large specific capacity and small voltage polarization, indicating strong adsorption of liquid polysulfides by SP and NOCF and rapid nucleation kinetics of solid Li_2_S (Figs. 4e, S16, and S17). As revealed by SEM and S mapping (Fig. 4f–h), the active materials of S in the SP/S/NOCF cathode are uniformly distributed between the fibers in the direction of the thickness at a high S loading of 6.0 mg cm^−2^. The SP/S/NOCF cathode (vs. Li foil) also shows much better cycling stability than that of the SP/S/CF and SP/S/GS cathodes owing to the dual-polarity design of SP and NOCF (Fig. 4i). The cycling stability of the sulfur cathode with various loadings from 2.6 to 6.0 mg cm^−2^ was also examined (Fig. 4j). At a high mass loading of 6.0 mg cm^−2^, the fabric SP/S/NOCF cathode exhibited a high areal capacity of 5.4 mAh cm^−2^ and maintained a high capacity retention of over 85% after 100 cycles. Therefore, a fabric sulfur cathode with high areal capacities and stable cycling performance is suitable for fabricating flexible Li–S batteries when paired with a fabric Li-metal anode.

### Cycling Stability and Flexibility of Lithium–Sulfur Full Batteries

**Fig. 5:**
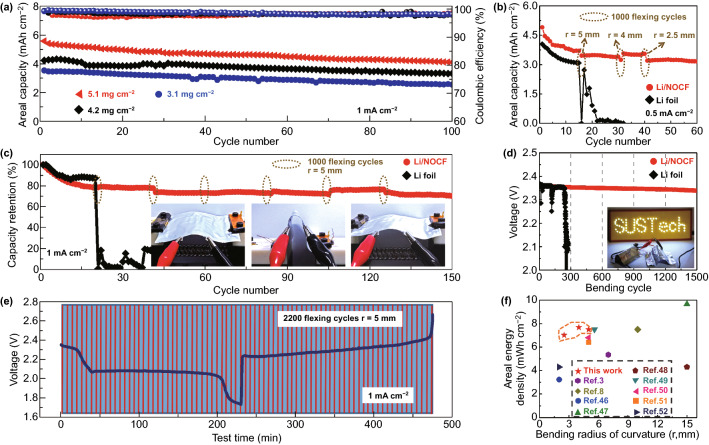
**a** Cycling stability of Li–S full cells (denoted as Li/NOCF//SP/S/NOCF) with various sulfur loadings of 5.1, 4.2, and 3.1 mg cm^−2^ along with 90% oversized Li. **b** Areal capacities of Li–S full cells with an electrode size of 6.0 cm^−2^ and high sulfur loadings of 3.9 mg cm^−2^ are recorded at 0.5 mA cm^−2^ for 60 cycles, during which 3000 flexing cycles are applied for Li//SP/S/NOCF and Li/NOCF//SP/S/NOCF at bending radii of 5.0, 4.0, and 2.5 mm. **c** Capacity retention of Li/NOCF//SP/S/NOCF full cells (S loading: 3.3 mg cm^−2^) is recorded during 6000 bending cycles at a bending radius of 5 mm. Insets are optical images of Li–S full cells during flexing process. **d** Voltage stability of Li–S full cells (S loading: 3.3 mg cm^−2^) during flexing process at a bending radius of 5 mm with a bending rate of 10 mm s^−1^. **e** Charge/discharge curves of Li/NOCF//SP/S/NOCF full batteries (S loading: 3.8 mg cm^−2^) during 2200 in situ flexing cycles with a bending rate of 10 mm s^−1^. **f** Figure of merit (FOM, *E*_a_/*r*) of Li/NOCF//SP/S/NOCF full cells and previously reported Li–S cells [3, 8, 46–52]

To demonstrate the cycling stability and mechanical flexibility of the fabric Li/NOCF anode and fabric SP/S/NOCF cathode, we stacked them with a polypropylene fabric to fabricate the full Li–S batteries. SP/S/NOCF cathodes with different sulfur loadings of 3.1, 4.2, and 5.1 mg cm^−2^ were paired with a limited amount of Li/NOCF anode (Fig. 5a). Importantly, both batteries with high and low mass loadings showed remarkable cell capacity and cycling stability. For example, the assembled fabric Li–S battery with a sulfur loading of 5.1 mg cm^−2^ and a limited Li excess of 90% exhibited large areal capacities of 5.6 mAh cm^−2^ and a high capacity retention rate of > 80% after 100 cycles. The excellent cycling stability of the cell is due to the structural integrity of both the Li and S electrodes during the charge/discharge process (Figs. S18-S22). Based on the total weight and volume of the entire cell, including the current collectors, electrodes, and separators, the Li–S full battery could provide high cell energy densities of 694.1 and 457.2 Wh L^−1^. Based on the total weight and volume of the pouch-cell model, including the current collectors, electrodes, separators, electrolytes, packaging materials, and metal tabs, the gravimetric and volumetric energy densities of Li–S full batteries were theoretically calculated to be 146.1 Wh kg^−1^ and 457.2 Wh L^−1^, respectively (Table S2). This result indicates that the future development of high-energy–density flexible Li–S full batteries requires not only the rational design of thin electrode materials, but also the use of limited Li excess and a lean electrolyte.

The fabric Li–S full battery is ideal for flexible and wearable applications. Here, various bending radii of 5.0, 4.0, and even 2.5 mm were applied when bending the fabric Li–S full cell (Fig. 5b). At a current density of 0.5 mA cm^−2^, no obvious capacity loss was observed for the fabric Li–S full cell (electrode size: 6.0 cm^−2^) during 3,000 bending cycles. At a practical current density of 1.0 mA cm^−2^, the cell could also maintain a high capacity retention of 75% during 6000 bending cycles (150 charge/discharge cycles) with a bending radius of 5.0 mm, indicating the excellent bending capability and stability of both fabric electrodes (Fig. 5c). In contrast, Li foil-based (100 µm) Li–S full batteries showed serious capacity fading after 1000 bending cycles at a bending radius of 5.0 mm owing to the structural damage and mechanical fracture of the Li foil anode.

To reach the voltage and stability requirements for industrial applications, two batteries with an electrode size of 6.0 cm^−2^ were connected in series to yield an open circuit voltage of 4.2 V and a high areal capacity of 4.8 mAh cm^−2^. The tandem cell was used to power a display screen of hundred LEDs (trigger voltage: 3.7 V; size: 25 × 10 cm^2^), where the logo of “SUSTech” was clearly shown. Moreover, the fabric Li–S full cell also showed negligible fluctuations of output voltage (less than 50 mV) during 1500 flexing cycles (Fig. 5d), which is in accordance with the stability requirements of industrial applications. In contrast, conventional Li foil-based Li–S pouch cells exhibited large voltage fluctuations and failed when bent 300 times (*r* = 5.0 mm), which is ascribed to the low fatigue resistance of the Li foil anode. To further investigate the mechanical stability of the fabric Li–S batteries, an in situ dynamic bending test of 2200 flexing cycles was carried out during the charge/discharge process at a bending rate of 10 mm s^−1^ (Fig. 5e). Overall, there is almost no shift in the charge/discharge curve, further indicating the excellent mechanical stability of the fabric Li–S full batteries. In earlier studies, most flexible Li–S batteries that were bent hundreds of times exhibited serious electrochemical instability during subsequent charge/discharge processes. Since the ratio (*E*_a_/*r*) of the areal energy density to the bending radius has been proposed as the figure of merit (FOM) for evaluating the performance of flexible batteries [39], we plotted an FOM chart comparing our fabric Li–S full batteries with previously reported flexible lithium batteries (Fig. 5f) [3, 8, 46–52]. As a result, the fabric Li–S full battery exhibited a much higher FOM than those of reported flexible lithium batteries, demonstrating its superior properties. To the best of our knowledge, such flexible Li–S batteries, with a unique material design and excellent electrochemical and mechanical properties, have not yet been reported.

## Conclusions

A cooperative strategy for stabilizing flexible Li–S full batteries with a limited Li excess of 90% has been proposed that employs silk fibroin/sericin to simultaneously inhibit lithium dendrites, adsorb dissolved polysulfides, and anchor solid lithium sulfides. The fabric Li–S full cell exhibited a high volumetric energy density (457.2 Wh L^−1^), excellent mechanical flexibility (6000 flexing cycles at 5 mm), and high capacity retention (99.8% per cycle). The excellent performance of the flexible Li–S full cell can be ascribed to the use of a stable Li anode and S cathode. The fabric structure of NOCF simultaneously endowed the electrodes with mechanical flexibility and reduced their local current density. On the anode side, NOCF rendered the uniform deposition of Li nanoparticles instead of dendrites and led to an average CE of > 99.4% over 300 charge/discharge cycles. On the cathode side, dual-polarity designed NOCF and PS cooperatively adsorbed dissolved polysulfides and promoted the uniform anchoring of solid Li_2_S, resulting in an excellent capacity retention of > 80% over 200 cycles. As a result, the trade-off between the electrochemical performance and mechanical flexibility of Li–S batteries was well resolved by the employment of rationally designed N/O-codoped fabrics. These materials and design principles can be applied to other flexible batteries (such as Li-ion batteries and Zn-metal batteries) to provide them with excellent mechanical flexibility, high energy density, and long cycling stability.

## Supplementary Information

Below is the link to the electronic supplementary material.

Supplementary file1 (PDF 1931 KB)

## References

[1] Chang J, Shang J, Sun Y, Ono LK, Wang D (2018). Flexible and stable high-energy lithium–sulfur full batteries with only 100% oversized lithium. Nat. Commun..

[2] Gao Y, Guo Q, Zhang Q, Cui Y, Zheng Z (2020). Fibrous materials for flexible Li–S battery. Adv. Energy Mater..

[3] Kim J-H, Lee Y-H, Cho S-J, Gwon J-G, Cho H-J (2019). Nanomat Li–S batteries based on all-fibrous cathode/separator assemblies and reinforced li metal anodes: towards ultrahigh energy density and flexibility. Energy Environ. Sci..

[4] Sun C, Wu T, Wang J, Li W, Jin J (2018). Favorable lithium deposition behaviors on flexible carbon microtube skeleton enable a high-performance lithium metal anode. J. Mater. Chem. A.

[5] Jiang C, Xiang L, Miao S, Shi L, Xie D (2020). Flexible interface design for stress regulation of a silicon anode toward highly stable dual-ion batteries. Adv. Mater..

[6] Zhu Y, Yang M, Huang Q, Wang D, Yu R (2020). V_2_O_5_ textile cathodes with high capacity and stability for flexible lithium-ion batteries. Adv. Mater..

[7] Zhao CX, Chen WJ, Zhao M, Song YW, Liu JN (2020). Redox mediator assists electron transfer in lithium–sulfur batteries with sulfurized polyacrylonitrile cathodes. EcoMat.

[8] Yi H, Yang Y, Lan T, Zhang T, Xiang S (2020). Water-based dual-cross-linked polymer binders for high-energy-density lithium–sulfur batteries. ACS Appl. Mater. Interfaces.

[9] Peng H-J, Huang J-Q, Cheng X-B, Zhang Q (2017). Review on high-loading and high-energy lithium–sulfur batteries. Adv. Energy Mater..

[10] Yuan H, Huang J-Q, Peng H-J, Titirici M-M, Xiang R (2018). A review of functional binders in lithium–sulfur batteries. Adv. Energy Mater..

[11] Liu J, Galpaya DGD, Yan L, Sun M, Lin Z (2017). Exploiting a robust biopolymer network binder for an ultrahigh-areal-capacity Li–S battery. Energy Environ. Sci..

[12] Razaq R, Zhang N, Xin Y, Li Q, Wang J (2020). Electrocatalytic conversion of lithium polysulfides by highly dispersed ultrafine Mo_2_C nanoparticles on hollow n-doped carbon flowers for Li–S batteries. EcoMat.

[13] Song N, Gao Z, Zhang Y, Li X (2019). B_4_C nanoskeleton enabled, flexible lithium–sulfur batteries. Nano Energy.

[14] Wang C, Xia K, Wang H, Liang X, Yin Z (2019). Advanced carbon for flexible and wearable electronics. Adv. Mater..

[15] Han B, Feng D, Li S, Zhang Z, Zou Y (2020). Self-regulated phenomenon of inorganic artificial solid electrolyte interphase for lithium metal batteries. Nano Lett..

[16] Pang Q, Liang X, Kochetkov IR, Hartmann P, Nazar LF (2018). Stabilizing lithium plating by a biphasic surface layer formed in situ. Angew. Chem. Int. Ed..

[17] Liu S, Ji X, Yue J, Hou S, Wang P (2020). High interfacial-energy interphase promoting safe lithium metal batteries. J. Am. Chem. Soc..

[18] Wang M, Peng Z, Luo W, Ren F, Li Z (2019). Tailoring lithium deposition via an sei-functionalized membrane derived from LiF decorated layered carbon structure. Adv. Energy Mater..

[19] Fang R, Xu H, Xu B, Li X, Li Y (2020). Reaction mechanism optimization of solid-state Li–S batteries with a PEO-based electrolyte. Adv. Funct. Mater..

[20] Peng HJ, Huang JQ, Zhang Q (2017). A review of flexible lithium–sulfur and analogous alkali metal-chalcogen rechargeable batteries. Chem. Soc. Rev..

[21] Luo L, Chung SH, Yaghoobnejad Asl H, Manthiram A (2018). Long-life lithium–sulfur batteries with a bifunctional cathode substrate configured with boron carbide nanowires. Adv. Mater..

[22] Chang C-H, Chung S-H, Manthiram A (2017). Highly flexible, freestanding tandem sulfur cathodes for foldable Li–S batteries with a high areal capacity. Mater. Horiz..

[23] Song X, Wang S, Bao Y, Liu G, Sun W (2017). A high strength, free-standing cathode constructed by regulating graphitization and the pore structure in nitrogen-doped carbon nanofibers for flexible lithium–sulfur batteries. J. Mater. Chem. A.

[24] Wang Z, Shen J, Liu J, Xu X, Liu Z (2019). Self-supported and flexible sulfur cathode enabled via synergistic confinement for high-energy-density lithium–sulfur batteries. Adv. Mater..

[25] Cheng Z, Pan H, Chen J, Meng X, Wang R (2019). Separator modified by cobalt-embedded carbon nanosheets enabling chemisorption and catalytic effects of polysulfides for high-energy-density lithium–sulfur batteries. Adv. Energy Mater..

[26] Yun JH, Kim JH, Kim DK, Lee HW (2018). Suppressing polysulfide dissolution via cohesive forces by interwoven carbon nanofibers for high-areal-capacity lithium–sulfur batteries. Nano Lett..

[27] Liu S, Li J, Yan X, Su Q, Lu Y (2018). Superhierarchical cobalt-embedded nitrogen-doped porous carbon nanosheets as two-in-one hosts for high-performance lithium–sulfur batteries. Adv. Mater..

[28] Li N, Zhang K, Xie K, Wei W, Gao Y (2020). Reduced-graphene-oxide-guided directional growth of planar lithium layers. Adv. Mater..

[29] Guan L, Hu H, Li L, Pan Y, Zhu Y (2020). Intrinsic defect-rich hierarchically porous carbon architectures enabling enhanced capture and catalytic conversion of polysulfides. ACS Nano.

[30] Li Q, Ma Z, Li J, Liu Z, Fan L (2020). Core–shell-structured sulfur cathode: ultrathin delta-MnO_2_ nanosheets as the catalytic conversion shell for lithium polysulfides in high sulfur content lithium–sulfur batteries. ACS Appl. Mater. Interfaces.

[31] Dong Y, Liu Y, Hu Y, Ma K, Jiang H (2020). Boosting reaction kinetics and reversibility in Mott–Schottky VS_2_/MoS_2_ heterojunctions for enhanced lithium storage. Sci. Bull..

[32] Sun Z, Zhang J, Yin L, Hu G, Fang R (2017). Conductive porous vanadium nitride/graphene composite as chemical anchor of polysulfides for lithium–sulfur batteries. Nat. Commun..

[33] Luo L, Li J, Yaghoobnejad Asl H, Manthiram A (2019). A 3d lithiophilic Mo_2_N-modified carbon nanofiber architecture for dendrite-free lithium-metal anodes in a full cell. Adv. Mater..

[34] Zhai P, Wang T, Yang W, Cui S, Zhang P (2019). Uniform lithium deposition assisted by single-atom doping toward high-performance lithium metal anodes. Adv. Energy Mater..

[35] Du Z, Chen X, Hu W, Chuang C, Xie S (2019). Cobalt in nitrogen-doped graphene as single-atom catalyst for high-sulfur content lithium–sulfur batteries. J. Am. Chem. Soc..

[36] Pathak R, Chen K, Gurung A, Reza KM, Bahrami B (2020). Fluorinated hybrid solid-electrolyte-interphase for dendrite-free lithium deposition. Nat. Commun..

[37] Chen X, Hou T, Persson KA, Zhang Q (2019). Combining theory and experiment in lithium–sulfur batteries: current progress and future perspectives. Mater. Today.

[38] Liu L, Yin Y-X, Li J-Y, Wang S-H, Guo Y-G (2018). Uniform lithium nucleation/growth induced by lightweight nitrogen-doped graphitic carbon foams for high-performance lithium metal anodes. Adv. Mater..

[39] Chang J, Huang Q, Zheng Z (2020). A figure of merit for flexible batteries. Joule.

[40] Wang C, Li X, Gao E, Jian M, Xia K (2016). Carbonized silk fabric for ultrastretchable, highly sensitive, and wearable strain sensors. Adv. Mater..

[41] Lu W, Jian M, Wang Q, Xia K, Zhang M (2019). Hollow core-sheath nanocarbon spheres grown on carbonized silk fabrics for self-supported and nonenzymatic glucose sensing. Nanoscale.

[42] Li X, Zhao J, Cai Z, Ge F (2018). A dyeing-induced heteroatom-co-doped route toward flexible carbon electrode derived from silk fabric. J. Mater. Sci..

[43] Li X, Sun C, Cai Z, Ge F (2019). High-performance all-solid-state supercapacitor derived from PPY coated carbonized silk fabric. Appl. Surf. Sci..

[44] Rocha LKH, Favaro LIL, Rios AC, Silva EC, Silva WF (2017). Sericin from bombyx mori cocoons. Part i: extraction and physicochemical-biological characterization for biopharmaceutical applications. Process Biochem..

[45] Park CJ, Ryoo J, Ki CS, Kim JW, Kim IS (2018). Effect of molecular weight on the structure and mechanical properties of silk sericin gel, film, and sponge. Int. J. Biol. Macromol..

[46] Wu C, Fu L, Maier J, Yu Y (2015). Free-standing graphene-based porous carbon films with three-dimensional hierarchical architecture for advanced flexible li–sulfur batteries. J. Mater. Chem. A.

[47] Chen J, Zhang H, Yang H, Lei J, Naveed A (2020). Towards practical Li–S battery with dense and flexible electrode containing lean electrolyte. Energy Storage Mater..

[48] Zhou G, Li L, Wang DW, Shan XY, Pei S (2015). A flexible sulfur-graphene-polypropylene separator integrated electrode for advanced Li-S batteries. Adv. Mater..

[49] Xiang M, Wu H, Liu H, Huang J, Zheng Y (2017). A flexible 3d multifunctional MgO-decorated carbon foam@cnts hybrid as self-supported cathode for high-performance lithium-sulfur batteries. Adv. Funct. Mater..

[50] Mao Y, Li G, Guo Y, Li Z, Liang C (2017). Foldable interpenetrated metal-organic frameworks/carbon nanotubes thin film for lithium–sulfur batteries. Nat. Commun..

[51] Xiao P, Bu F, Yang G, Zhang Y, Xu Y (2017). Integration of graphene, nano sulfur, and conducting polymer into compact, flexible lithium–sulfur battery cathodes with ultrahigh volumetric capacity and superior cycling stability for foldable devices. Adv. Mater..

[52] Yu M, Wang Z, Wang Y, Dong Y, Qiu J (2017). Freestanding flexible Li_2_S paper electrode with high mass and capacity loading for high-energy Li–S batteries. Adv. Energy Mater..

