# Supplementary material for: Natural Cocoons Enabling Flexible and Stable Fabric Lithium–Sulfur Full Batteries
Source: Nanomicro Lett. 2021 Mar 6;13:84. doi: 10.1007/s40820-021-00609-3 (PMC8006205; doi:10.1007/s40820-021-00609-3)
Supplement: Supplementary file 1 — Supplementary file1 (PDF 1931 KB) [file 40820_2021_609_MOESM1_ESM.pdf]

Supporting Information for

Natural Cocoons Enabling Flexible and Stable Fabric Lithium-Sulfur Full Batteries

Yanan An[1], Chao Luo[2], Dahua Yao[1], Shujing Wen[2], Peitao Zheng[2], Shangsen Chi[2], Yu Yang[3], Jian Chang[2, 4, *], Yonghong Deng[2, 4, *], Chaoyang Wang[1, *]

[1]Research Institute of Materials Science, South China University of Technology, Guangzhou 510640, P. R. China

[2]Department of Materials Science & Engineering, Guangdong Provincial Key Laboratory of Energy Materials for Electric Power, Southern University of Science and Technology, Shenzhen 518055, P. R. China

[3]College of Materials and Energy, Guangdong Laboratory for Lingnan Modern Agriculture, South China Agricultural University, Guangzhou, 510642, P. R. China.

[4]Academy for Advanced Interdisciplinary Studies, Southern University of Science and Technology, Shenzhen 518055, P. R. China

*Corresponding authors. E-mail: changj@sustech.edu.cn (J Chang); yhdeng08@163.com (YH Deng); zhywang@scut.edu.cn (CY Wang)

Supplementary Tables and Figures

Table S1 Sheet resistance of NOCF samples under various annealing temperatures

Carbonization temperature (°C)	Sheet resistance (Ω cm^{-2})
1000	8.9
800	73.8
600	~10^3
400	~10^4

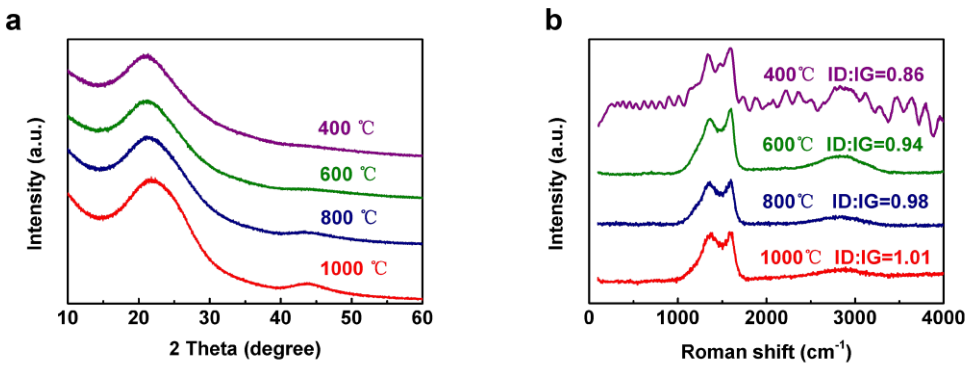

Fig. S1 a XRD pattern and **b** Raman spectra of NOCFs under different annealing temperatures

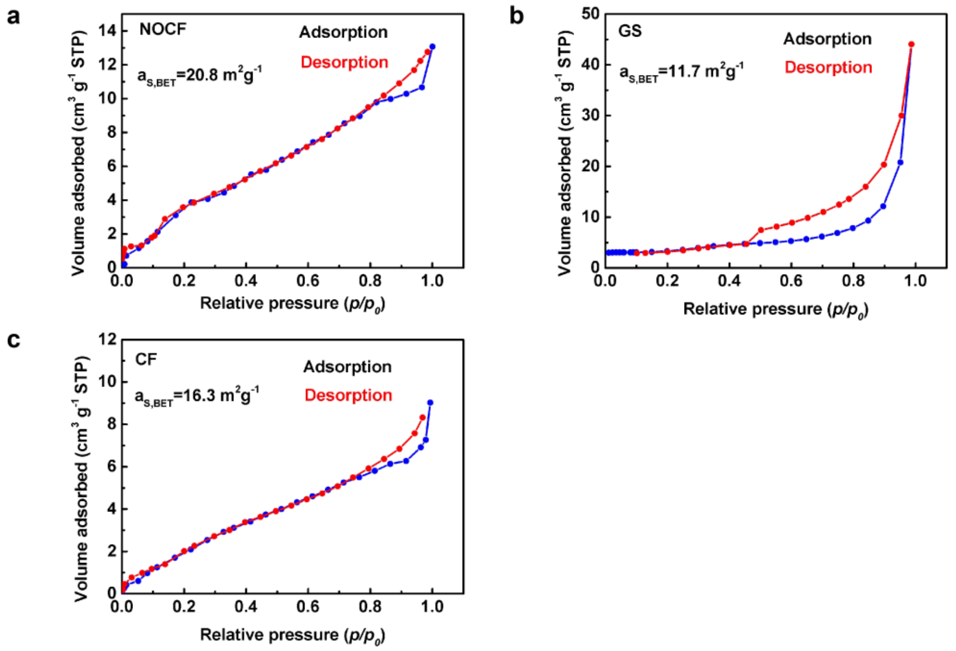

Fig. S2 Nitrogen adsorption–desorption isotherms of NOCF, CF, and GS

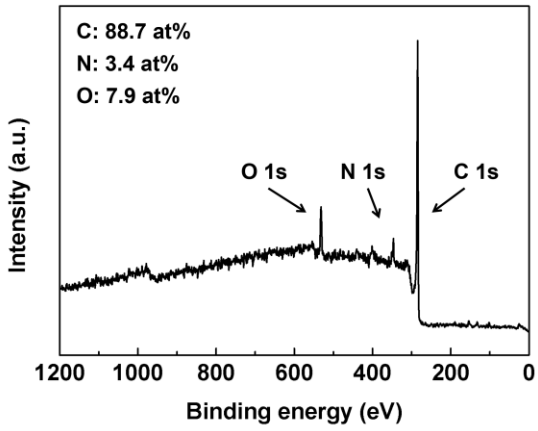

Fig. 3 XPS survey spectrum of NOCF carbonized at 1000 ºC

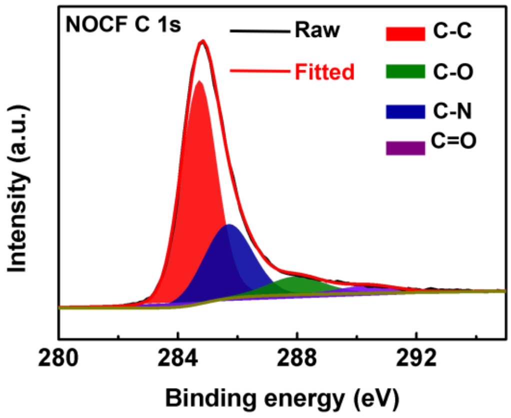

Fig. 4 High resolution XPS spectrum of C 1s for optimal NOCF

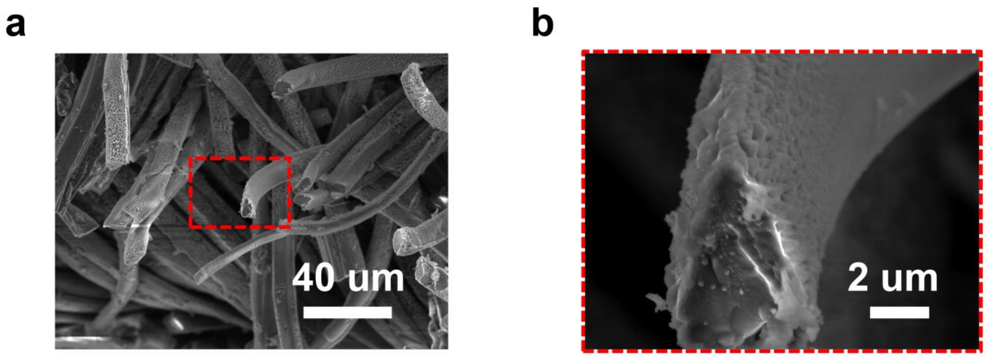

Fig. 5 a, b SEM cross sections of NOCF after plating 3 mAh cm^{-2} of Li at 1.0 mA cm^{-2}

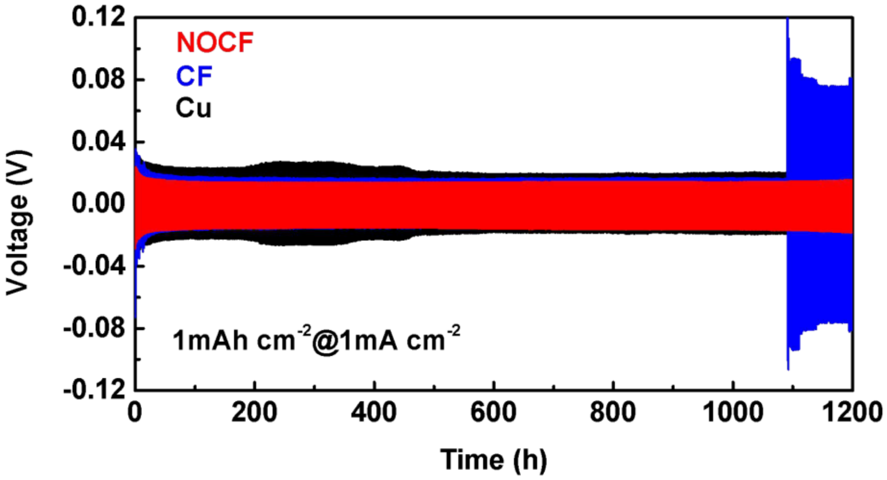

Fig. 6 Galvanostatic plating/stripping profiles of symmetric cells with different Li electrodes at 1.0 mA cm^{-2} for 1.0 mAh cm^{-2}

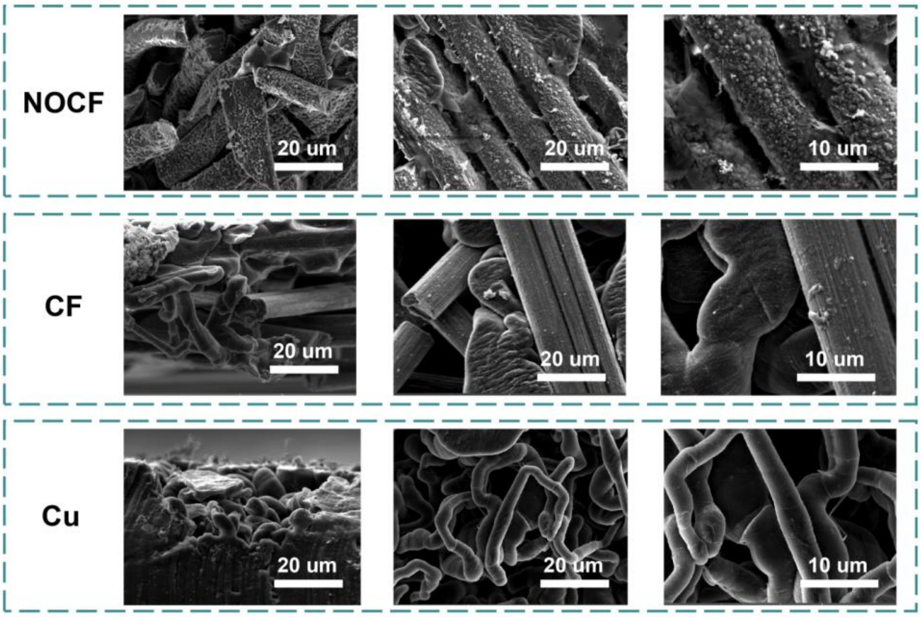

Fig. 7 SEM images of different current collectors (NOCF, CF and Cu) after plating 6 mAh cm^{-2} of Li at 1.0 mA cm^{-2}.

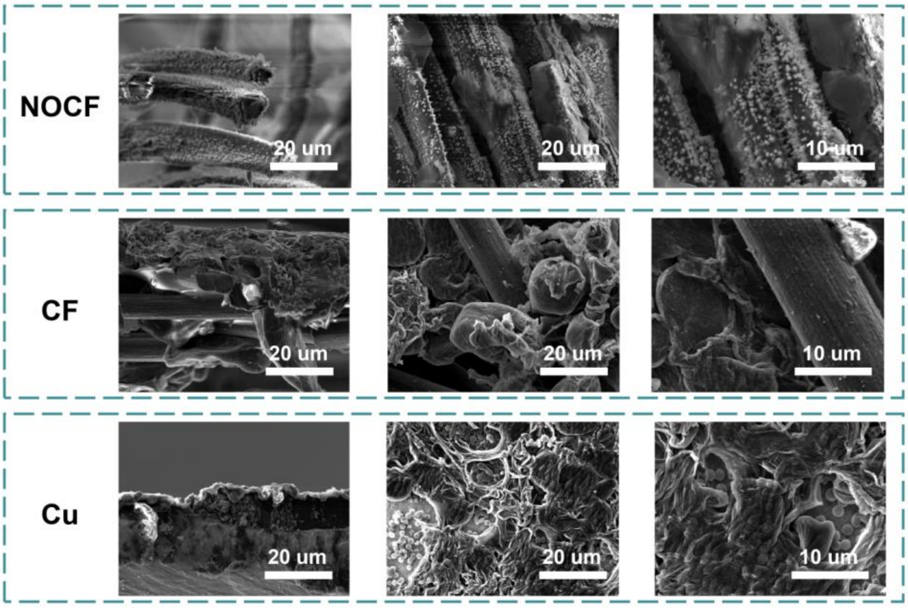

Fig. 8 SEM images of different current collectors (NOCF, CF and Cu) after plating 6 mAh cm^{-2} of Li and stripping 3.0 mAh cm^{-2} of Li at 1.0 mA cm^{-2}

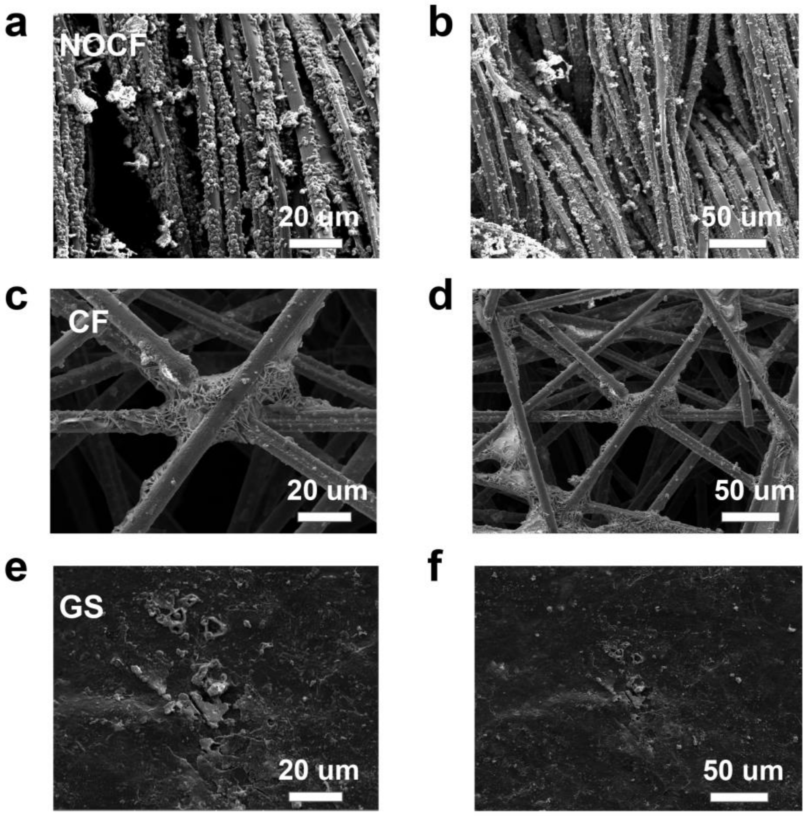

Fig. 9 Low magnification SEM images of Li_2S deposition on the surface of different current collectors (**a, b** NOCF, **c, d** CF and **e, f** GS) after discharging at 0.25 mA cm^{-2}

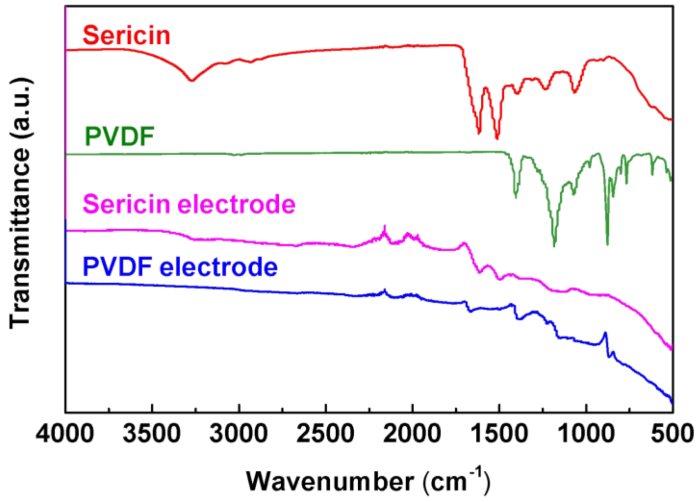

Fig. 10 FTIR spectra of SP (sericin protein), PVDF and corresponding S cathodes

The SP curve shows the typical adsorption peaks of sericin protein: 1624 cm^{-1} (amide I band 1600-1700 cm^{-1}), 1523 cm^{-1} (amide II band 1504-1582 cm^{-1}), 1232 cm^{-1} (amide III band 1200-1300 cm^{-1}) [S1, S2]. The broad peaks at 3600-3200 cm^{-1} correspond to the stretching vibration of -OH. These adsorption peaks indicate that SP is rich in hydroxyl, amino, amide and other polar functional groups. It can be seen from the figure that the FTIR curve of the sulfur cathode with SP as the binder retains the above peaks. These polar functional groups can adsorb polysulfides and inhibit the shuttle effect of Li-S batteries.

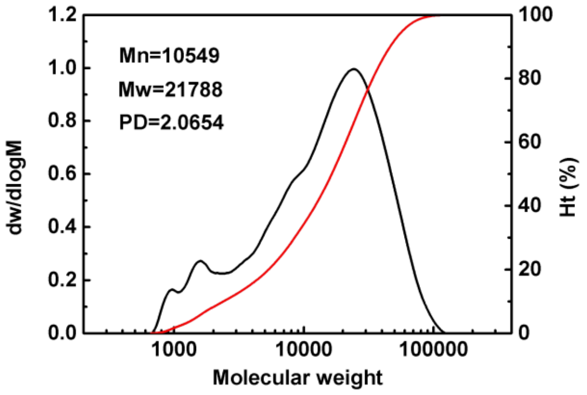

Fig. 11 Molecular weight distribution of sericin protein

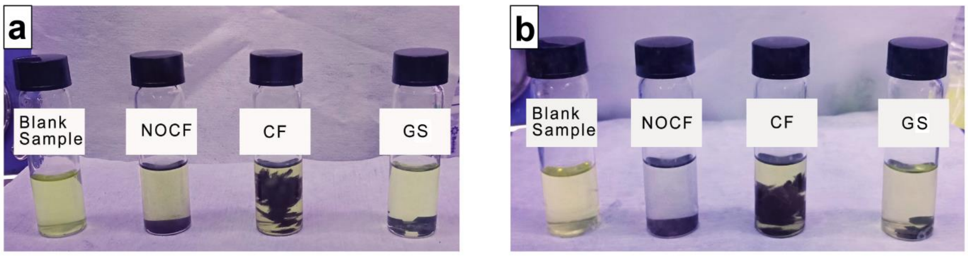

Fig. 12 a, b Photographs of Li_2S_8 solution before (**a**) and after (**b**) the addition of the same mass of sericin protein and PVDF

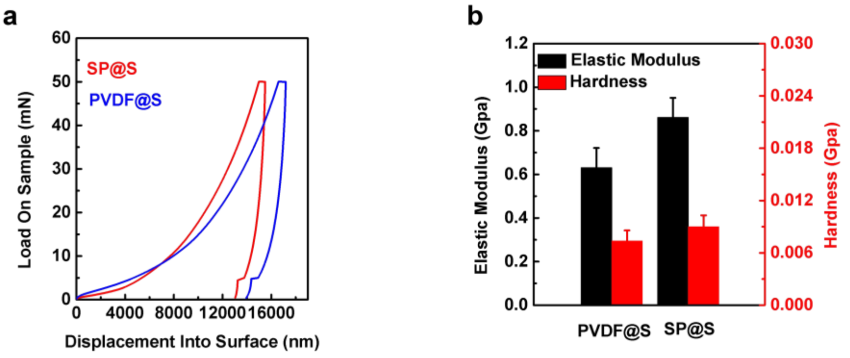

Fig. 13 Nanoindentation test for different sulfur cathodes. **a** typical load-indentation depth curve of SP- or PVDF-based sulfur cathodes. **b** Hardness and elastic modulus of SP-or PVDF-based sulfur cathodes

The Keysight UTM150 nanoindentation system was used to conduct a nanoindentation test on the sulfur cathodes with a Continuous Stiffness Measurement indentation method to evaluate the adhesion capability of the binder and structural stability of the electrodes [S3].

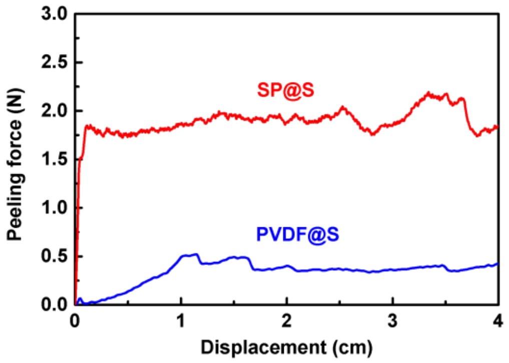

Fig. 14 Peeling force-displacement curves of SP- and PVDF-based sulfur cathode

Electrode samples (8 mm×50 mm) with different binders were firstly attached to the 3M tape, while backsides (Al foil) of the electrodes were paste on double-sided adhesive tape [S4, S5]. The whole piece was then fixed on a glass slide, and peel force of electrode samples was measured with a high-precision tensile test system (Instron 3342). The applied load was continuously recorded with the constant pulling rate of 500 μm s^{-1}.

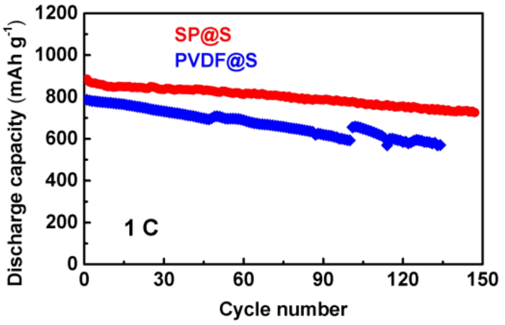

Fig. 15 Cycling performance of SP and PVDF based S cathodes at 1C (S loads=1.2-1.5 mg cm^{-2}, the current collector is aluminum foil)

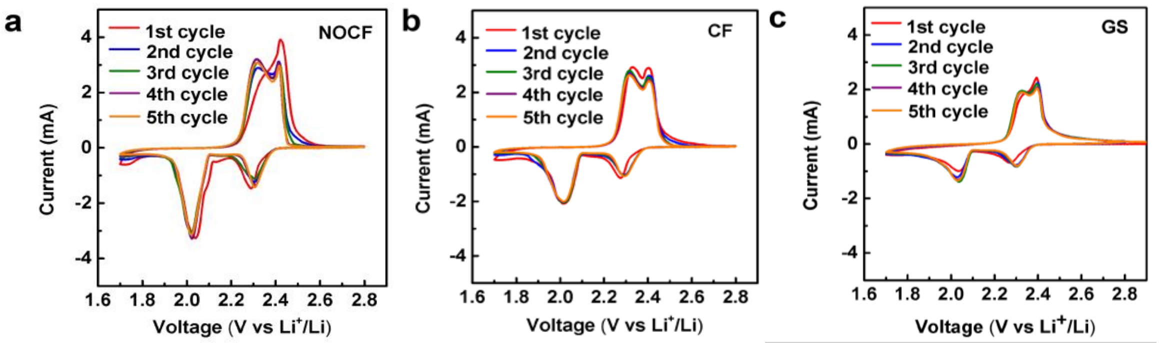

Fig. 16 Typical CV profiles of (**a**) SP/S/NOCF, (**b**) SP/S/CF and (**c**) SP/S/GS electrodes at initial five cycles

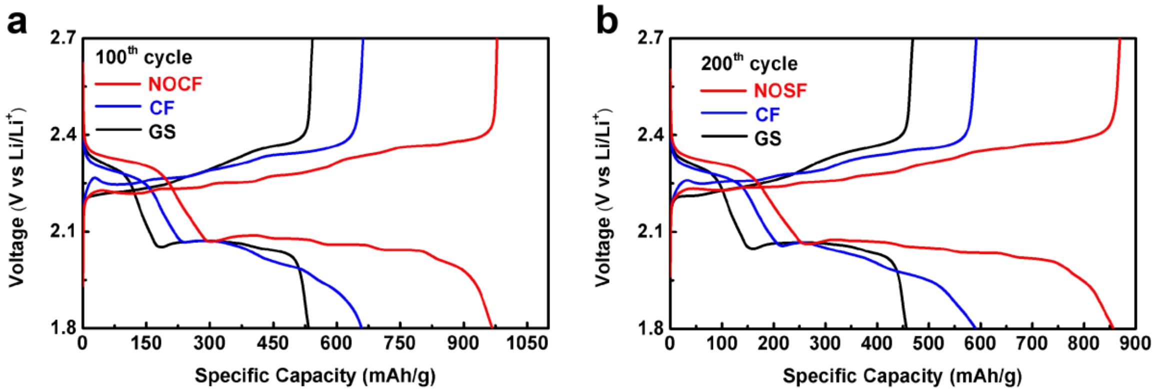

Fig. 17 Charge/discharge profiles of various S cathodes (SP/S/NOCF, SP/S/CF and SP/S/GS) for **a** 100th and **b** 200th cycles at 1 mA cm^{-2}

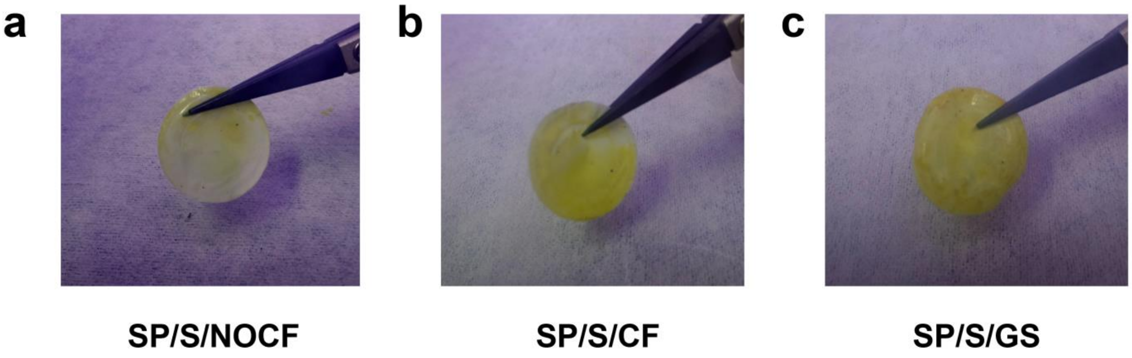

Fig. 18 Camera images of the separators extracted from different lithium-sulfur half-cells after 20 charge-discharge cycles (S loading = 3.0 mg cm^{-2})

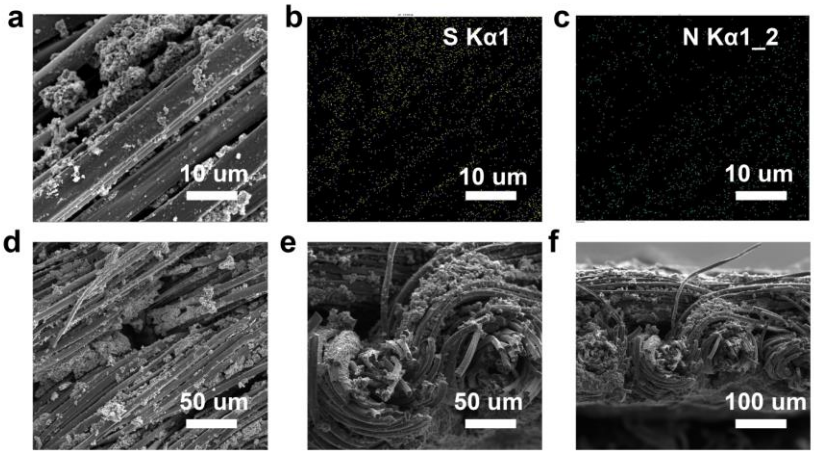

Fig. 19 a High-resolution top-view SEM images of SP/S/NOCF cathode after 20 cycles at 1 mA cm^{-2}. **b, c** Corresponding EDS mapping. **d** Low-resolution top-view SEM images of SP/S/NOCF cathode after 20 cycles at 1 mA cm^{-2}. **e, f** Cross-sectional SEM images of SP/S/NOCF cathode after 20 cycles at 1 mA cm^{-2}

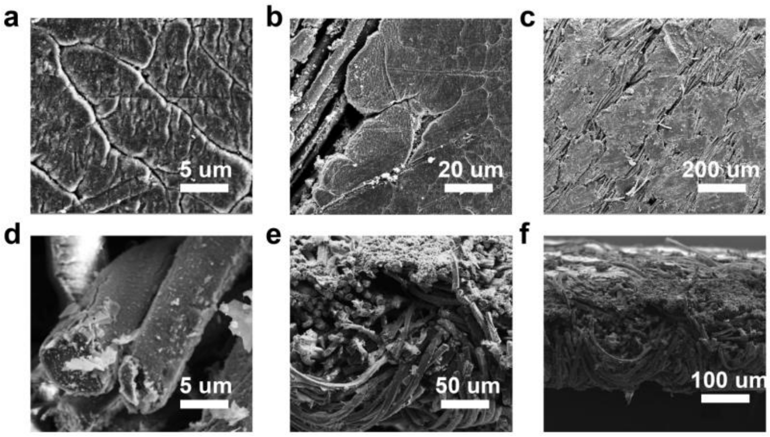

Fig. 20 a-f SEM images of Li/NOCF anode after 20 cycles at 1 mA cm^{-2}

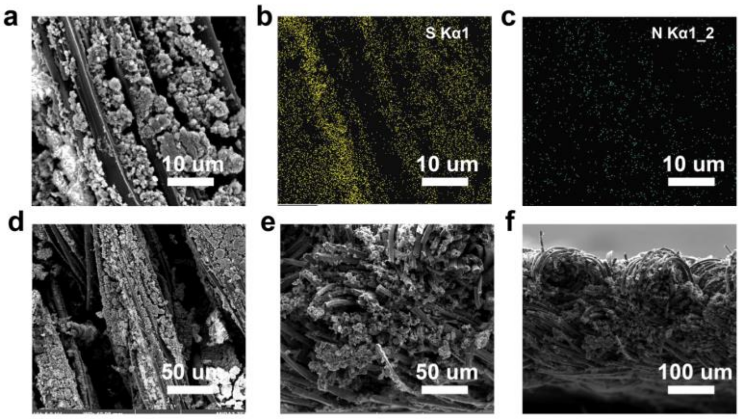

Fig. 21 a High-resolution top-view SEM images of SP/S/NOCF cathode after 50 cycles at 1 mA cm^{-2}. **b, c** corresponding EDS elemental mapping. **d** Low-resolution top-view SEM images of SP/S/NOCF cathode after 50 cycles at 1 mA cm^{-2}. **e, f** Cross-sectional SEM images of SP/S/NOCF cathode after 50 cycles at 1 mA cm^{-2}

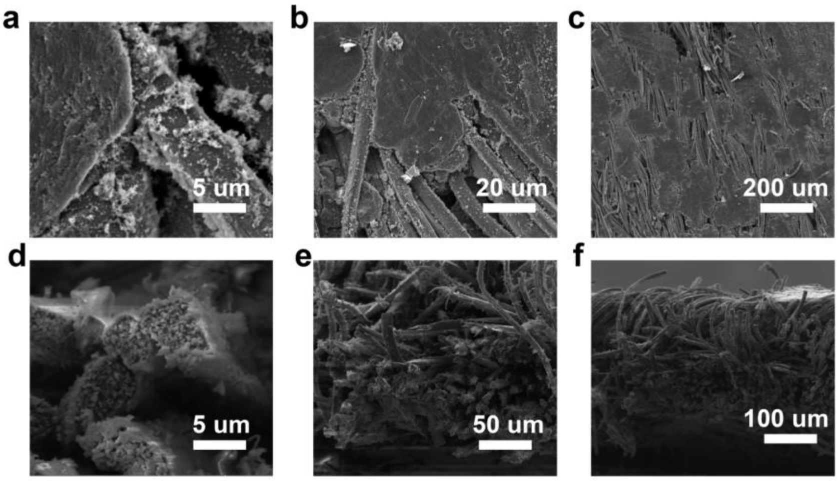

Fig. 22 a-f SEM images of Li/NOCF anode after 50 cycles at 1 mA cm^{-2}

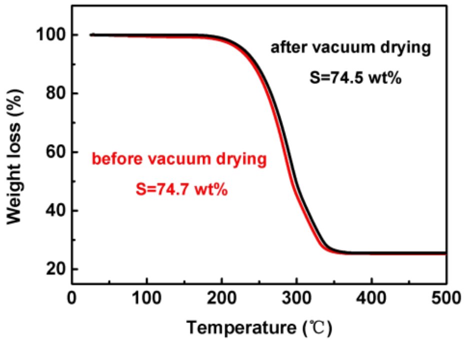

Fig. 23 TGA curves of S/C composite materials

TGA tests are performed for the S/C composites before and after vacuum drying at 60 °C for 12 hours. It can be seen from the experimental results that the sulfur content of the materials before and after drying is close (~75 wt%), indicating that the sublimation phenomenon of sulfur can be ignored.

Table S2 Critical parameters of our fabric Li-S full batteries for calculating cell energy densities

Sulfur loading (mg cm^{-2})	Cathode thickness (μm)	Cathode density (mg cm^{-2})	Cathode mass density (g cm^{-3})	Anode thickness (μm)	Anode density (mg cm^{-2})	Anode mass density (g cm^{-3})	Areal capacity (mAh cm^{-2})	Areal energy (mWh cm^{-2})
5.1	217	10.4	0.48	195	5.5	0.28	5.6	11.8
4.2	210	9.0	0.43	195	5.5	0.28	4.4	9.2
3.1	202	7.4	0.37	195	5.5	0.28	3.6	7.6

Noted, the thickness of the NOCF current collector for assembling Li-S full cells is 185 μm and the areal mass is 5.6 mg cm^{-2}. The Li/NOCF anode contains 10 mAh cm^{-2} of lithium (2.7

mg cm^{-2} for Li metal). To increase the areal capacity and energy density of Li-S full batteries, the SP/S/NOCF cathode (weight ratio of electrode materials: S: Ketjen black: SP binder= 67.6:12.5:10) with various S loading of 5.1, 4.2, and 3.1 mg cm^{-2} is prepared to pair with the Li/NOCF anode. The electrolyte/sulfur (E/S) ratio is 10 µl mg^{-1} and the electrolyte density of 1M LiTFSI in DOL/DME (2 wt% $LiNO_3$) is 1.1 g cm^{-3}. The thickness of the used separator (Celgard 2500) is 25 µm and the areal mass is measured to be 1.1 mg cm^{-2}.

The gravimetric and volumetric energy density of fabric Li-S full batteries are calculated based on the following equations:

$$E_g = \frac{VC}{\sum m_i} \qquad \text{(S1)}$$

$$E_v = \frac{VC}{\sum t_i} \qquad \text{(S2)}$$

Where E_g and E_v are the full-cell gravimetric (Wh kg^{-1}) and volumetric (Wh L^{-1}) energy densities, respectively. V is the nominal output voltage (V), C is the electrode areal capacity (mAh cm^{-2}), m_i is the total areal mass of cell components and t_i is the total thickness (µm) of cell components.

The gravimetric and volumetric energy densities of fabric Li-S full cells are calculated according to the specific cell components, as shown below:

1. Based on the total weight and volume of the entire cell including current collectors, electrode and separator:

$$E_g = \frac{VC}{\sum m_i} = \frac{VC}{m_{anode} + m_{cathode} + m_{separator}}$$

$$= \frac{2.1\ \text{V} \times 5.6\ \text{mAh cm}^{-2}}{(5.6 \div 2 + 2.7)\text{mg cm}^{-2} + (5.6 \div 2 + 5.1 \div 0.675)\text{mg cm}^{-2} + 1.1\ \text{mg cm}^{-2}}$$

$$= \frac{2.1\ \text{V} \times 5.6\ \text{mAh cm}^{-2}}{5.5\ \text{mg cm}^{-2} + 10.4\ \text{mg cm}^{-2} + 1.1\ \text{mg cm}^{-2}} = 694.1\ \text{Wh kg}^{-1}$$

$$E_v = \frac{VC}{\sum t_i} = \frac{VC}{t_{anode} + t_{cathode} + t_{separator}} = \frac{2.1\ \text{V} \times 5.6\ \text{mAh cm}^{-2}}{(195 \div 2)\mu\text{m} + (217 \div 2)\mu\text{m} + 25\ \mu\text{m}}$$

$$= \frac{2.1\ \text{V} \times 5.6\ \text{mAh cm}^{-2}}{97.5\ \mu\text{m} + 108.5\ \mu\text{m} + 25\ \mu\text{m}} = 510.8\ \text{Wh L}^{-1}$$

2. Based on the total weight and volume of the entire cell including current collectors, electrode, separator and electrolyte:

$$E_g = \frac{VC}{\sum m_i} = \frac{VC}{m_{anode} + m_{cathode} + m_{separator} + m_{electrolyte}}$$

$$= \frac{2.1\ \mathrm{V} \times 5.6\ \mathrm{mAh\ cm^{-2}}}{5.5\ \mathrm{mg\ cm^{-2}} + 10.4\ \mathrm{mg\ cm^{-2}} + 1.1\ \mathrm{mg\ cm^{-2}} + 56.1\ \mathrm{mg\ cm^{-2}}}$$

$$= 161.4\ \mathrm{Wh\ kg^{-1}}$$

$$E_v = \frac{VC}{\sum t_i} = \frac{VC}{t_{anode} + t_{cathode} + t_{separator}} = \frac{2.1\ \mathrm{V} \times 5.6\ \mathrm{mAh\ cm^{-2}}}{(195 \div 2)\mu\mathrm{m} + (217 \div 2)\mu\mathrm{m} + 25\ \mu\mathrm{m}}$$

$$= \frac{2.1\ \mathrm{V} \times 5.6\ \mathrm{mAh\ cm^{-2}}}{97.5\ \mu\mathrm{m} + 108.5\ \mu\mathrm{m} + 25\ \mu\mathrm{m}} = 510.8\ \mathrm{Wh\ L^{-1}}$$

Noted, the cell volume is unchanged after injecting electrolyte due to the full adsorption of electrolyte into the fabric electrode and separator.

3. Based on the total weight and volume of the entire cell including current collectors, electrode, separator, electrolyte, packaging materials and metal tabs:

$$E_g = \frac{VC}{(m_{anode} + m_{cathode} + m_{separator} + m_{electrolyte}) \div (1 - 9.5\%)}$$

$$= \frac{2.1\ \mathrm{V} \times 5.6\ \mathrm{mAh\ cm^{-2}}}{(5.5 + 10.4 + 1.1 + 56.1)\mathrm{mg\ cm^{-2}} \div (1 - 9.5\%)} = 146.1\ \mathrm{Wh\ kg^{-1}}$$

$$E_v = \frac{VC}{(t_{anode} + t_{cathode} + t_{separator}) \div (1 - 10.5\%)}$$

$$= \frac{2.1\ \mathrm{V} \times 5.6\ \mathrm{mAh\ cm^{-2}}}{(97.5 + 108.5 + 25)\ \mu\mathrm{m} \div (1 - 10.5\%)} = 457.2\ \mathrm{Wh\ L^{-1}}$$

Noted, the mass ratio (9.5 %) and thickness ratio (10.5 %) of packaging materials and metal tabs should be adopted for fairly comparing single-pair electrode pouch cell with tandem pouch cells, as mentioned in previous reports [6,7].

Supplementary References

[S1] L. K. H. Rocha, L. I. L. Favaro, A. C. Rios, E. C. Silva, W. F. Silva et al., Sericin from bombyx mori cocoons. Part i: Extraction and physicochemical-biological characterization for biopharmaceutical applications. Process Biochem. **61,** 163-177 (2017). https://doi.org/ 10.1016/j.procbio.2017.06.019

[S2] C. J. Park, J. Ryoo, C. S. Ki, J. W. Kim, I. S. Kim et al., Effect of molecular weight on the structure and mechanical properties of silk sericin gel, film, and sponge. Int. J. Biol. Macromol. **119**, 821-832 (2018). https://doi.org/10.1016/j.ijbiomac.2018.08.006

[S3] Y. Tang, J. Deng, W. Li, O. I. Malyi, Y. Zhang et al., Water-soluble sericin protein enabling stable solid-electrolyte interphase for fast charging high voltage battery

electrode. Adv. Mater. **29**, 33 (2017). https://doi.org/10.1002/adma.201701828

[S4] J. Liu, D. G. D. Galpaya, L. Yan, M. Sun, Z. Lin et al., Exploiting a robust biopolymer network binder for an ultrahigh-areal-capacity Li-S battery. Environ. Sci. **10**(3), 750-755 (2017). https://doi.org/10.1039/c6ee03033e

[S5] X. Fu, L. Scudiero, W.-H. Zhong, A robust and ion-conductive protein-based binder enabling strong polysulfide anchoring for high-energy lithium–sulfur batteries. J. Mater. Chem. A **7**(4), 1835-1848 (2019). https://doi.org/10.1039/c8ta11384j

[S6] Y. Gao, M. Guo, K. Yuan, C. Shen, Z. Ren, et al., Multifunctional silanization interface for high-energy and low-gassing lithium metal pouch cells. Adv. Energy Mater. **10**(4), (2019). https://doi.org/10.1002/aenm. 201903362

[S7] C. Niu, H. Lee, S. Chen, Q. Li, J. Du et al., High-energy lithium metal pouch cells with limited anode swelling and long stable cycles. Nat. Energy **4**(7), 551-559 (2019). https://doi.org/10.1038/s41560-019-0390-6
